# Ocular Lesions in Scarlatina

**Published:** 1904-09

**Authors:** 


### OCULAR LESIONS IN SCARLATINA.*

The ocular lesions that occur in scarlatina are of particular interest, for the reason that they are generally of a more or less serious nature. Scarlet fever, unlike diphtheria, is a disease that the average specialist does not come in contact with. It is handled in nearly every case by the general practitioner, and it is only when the eye symptoms are of a prominent character that a specialist is called in, and as the result the connection between many eye diseases and scarlet fever is lost sight of. Among the rarer lesions we have uremic amaurosis, purulent inflammation of the vitreous, phlegmon of the orbit, and thrombosis of the cavernous sinus.

In uremic amaurosis, which occurs more frequently than the other lesions, the blindness comes on suddenly and gets to be complete within a few hours or a day. After one or more days the sight is gradually restored.

* Presented by Ellet O. Sisson, M.D., Keokuk, Ia., member Ninth International Ophthalmological Congress, at the twelfth annual meeting of the Tri-State Medical Society, held at St. Louis, Mo., June 15, 16, 17, 1904.

Simultaneously with the attack of visual disturbance other nervous symptoms exist, such as headache, vomiting, dyspnea, loss of consciousness and convulsions. The fact that the reaction of the pupil to light is in most cases preserved in spite of the complete blindness proves that the location of the affection can not be in the eye, or in the optic nerve, but higher up—that is, in the brain, which is poisoned by the excretory matters contained in the blood. Cases are reported by Ebert (1), Forster (2), Monod (3), Power (4), Loeb (5), and Martin (6). These cases are quoted by Forster (7), who calls attention to the fact that in all of them albuminuris was present, and that the amaurosis occurred in the desquamation stage after a period of generally favorable symptoms. The amaurosis was ushered in by cerebral symptoms, headache, convulsions, vomiting and stupor. It came on suddenly, was bilateral, and for a time was complete. No ophthalmoscopic lesions were detected, and the blindness gradually cleared off.

Purulent inflammation of the vitreous or suppurative hyalitis is the product of a metastatic choroiditis, which sometimes follows in the wake of scarlet fever. In this lesion of the cornea is clear, a yellowish reflex is seen shining through the pupillary space, there is retraction of the periphery of the iris, and bulging of its pupillary border. Usually one or two synechiæ are present, and the tension is diminished. In addition to this, there may be a pericorneal zone of congestion, connected with the inflammation of the iris and ciliary body.

When the pus in the retina is circumscribed, the symptoms at the first glance are not unlike those of glioma of the retina, and the name pseudo-glioma has been given to this condition, especially as it is seen in children.

In phlegmon of the orbit, we have an inflammation of the cellulo-fatty tissue surrounding the eyeball. It is of metastatic origin and is generally of the acute form. It is usually monolateral, although it may be bilateral. Constitutional symptoms generally accompany it, such as chills and fever, and not infrequently, cerebral symptoms, such as headache, vomiting, mental hebetude, retardation of the pulse, etc. The local symptoms are exophthalmos, limitations in the movements of the eye, and swelling and edema of the lids, together with hyperemia and chemosis of the conjunctiva. When the symptoms have reached their acme, the skin of the lids at a certain spot grows red, then shows a yellow discoloration, and finally is perforated by a discharge of pus. After the evacuation of the pus, which is present in large quantity, the inflammatory symptoms in most cases rapidly subside and the opening heals again. The sight may suffer permanent diminution, or be altogether annihilated, if the optic nerve is implicated; for inflammation of the optic nerve or thrombosis of its vessels may develop, succeeded by atrophy of the nerve. Detachment of the retina, and even panophthalmitis, also occasionally occurs in retrobulbar phlegmon. If the suppuration is carried over from the orbit to the cranial cavity, it leads to a fatal issue through purulent meningitis or abscess of the brain.

In thrombosis of the cavernous sinus, we have a lesion which, from the pathologist's viewpoint, is of particular interest. That the lesion is a rare one is shown by the literature on the subject, there having been only 182 cases, the result of various causes reported up to date. Of this number only fourteen recovered. The lesion is set up metastatically. The symptoms are very prominent, and are similar to those which present themselves in the beginning of a retrobulbar phlegmon. The lids and the conjunctiva swell up with edema, and the eyeball is protruded and becomes hard to move. The veins of the retina are seen upon ophthalmoscopic examination to be distended enormously with blood. These symptoms are referable to the fact that the veins of the orbit discharge the greater part of their blood through the opthalmic veins into the cavernous sinus; if the latter is occluded, an extreme degree of venous stasis in the orbit necessarily takes place and leads to protrusion of the eyeball, and also venous hyperemia of the retina. This edema depends upon the fact that in this region an emissary vein of Santorini empties into the transverse sinus, and thus indirectly into the cavernous sinus, so that when there is occlusion of the latter this region also shares in the venous stasis. When this edema is present, it forms an important diagnostic sign between thrombosis of the cavernous sinus and retrobulbar phlegmon, in which latter it is absent. A further point of difference lies in the fact that thrombosis of the sinus frequently passes over to the other side, so that the same complex of symptoms develops there also, while, on the contrary, a bilateral orbital phlegmon would be one of the greatest rarities.

Wells and Germain (8) report a case that was operated upon, the cavernous sinus exposed, incised and drained. The patient died, but they claim that by the operation they demonstrated that the sinus is not inaccessible, that it may be reached without grave danger to the patient, and at least a low mortality from the operation itself, that it can be done under almost primary anesthesia, not associated with any degree of shock, finished within a few minutes—in their case eight—and that the hemorrhage is easily controlled. They also claim that an incision into one sinus instantly and completely relieved the interference with the circulation in both.

Retinitis albuminuria occurs after scarlatina, but it is not frequent; it is more a complication of the chronic form of Bright's disease than of the croupous nephritis which is found in scarlatina. The prognosis is more favorable than in albuminuric retinitis unconnected with an exanthem, but partial optic atrophy has been observed (9).

The sight can also be affected as the result of scarlatina without the presence of albuminuria, or any evidence of any kidney disease, as in the case recorded by Hodges (10), where thrombosis of the cerebral artery was observed in one eye of a growing girl while recovering from scarlatina. In this case the urine was normal, so that the ocular lesion may possibly be attributed to debility iuduced by the disease.

Pfluger (11) has observed papillo-retinitis after scarlatina without kidney affection, and the same phenomenon has been observed by Betke (12), though in the latter case the patient had suffered at an earlier period from hemiplegia. Leber (13) reports a case of a boy who became blind without ophthalmoscopic signs and with normal urine. The only assignable cause was latent scarlatina, and Leber seems to regard this peculiar case as somewhat analogous to post-diphtheritic lesions in other nerves.

Occasionally accommodative asthenopia shows itself after scarlatina as it does after measles. It may persist for a long period of time even after the general health is completely re-established. Forster instances the case of a boy of nine years who suffered from caries of both temporal bones and complete paralysis of both facial nerves. Both corneas were destroyed in consequence of the lagophthalmus, and the unfortunate patient became blind as well as deaf. Fuchs, in his text-book, states that suppurative choroiditis of metastatic origin may occur in scarlatina, as in typhus, variola, etc. In most, if not all of the recorded cases, suppurative otitis has preceded the choroiditis (or retinitis).

Phillips (14) has observed an edema of the upper eyelid during scarlatina, which also is apparently associated with suppurative otitis. The swelling was not white and doughy as in renal dropsy, but tense and livid, and in the cases recorded it was more marked before by rupture of the membrana tympani, and increased afterwards if the discharge from the ear became less free. Phillips considers it probable that the affection is connected with thrombosis of the cavernous sinus.

As the result of this brief study of the eye lesions that occur in scarlatina, we are justified in arriving at the following conclusions:

First. In view of the fact that scarlet fever is one of the most common of the exanthemata, and that the majority of eye lesions, occurring in connection with it, are of a serious nature, or involving not only loss of vision, but in some cases life itself, greater attention should be given them than they generally receive.

Second. That if the operation on the cavernous sinus can be made without grave danger to the patient, and with the chance of lessening the mortality, as claimed by the operators, such an operation is justifiable and should be performed.

BIBLIOGRAPHY.

1. Forster, Graefe und Saemisch's Handbuch der gesammten Augenheilkunde, Bd. VII, S. 162.

2. Forster, R., Scarlach mit nachfolgendes Nierenerkrankung und transitorische Erblindung, from Jahrbuch fur Kinderheilkunde und physische Erziehung in Klinische Monatsblatter fur praktische Augenheilkunde, 1872, S. 346.

3. Monod, Albuminuric aigne consecutive a la scarlatina, convulsions, epileptiformes, amaurose, guerison. Gazette des Hopitaux, 1870, p. 113; Jahresbericht der Ophthalmolgie von Nagel, 1870, S. 370.

4. Power, Case of Complete but Temporary Loss of Vision in an Attack of Scarlet Fever, Practitioner, May, p. 257; Jahresbericht der Ophthalmolgie von Nagel, 1871, S. 341.

5. Loeb, Transitorische Erblindung und persistirende Paralyse nach Scarlach Jahrbuch fur Kinderheilkunde, Bd. VIII, S. 194; Forster, loco citato.

6. Martin, St. Bartholomew's Hospital Reports, 1867, p. 246. Temporary Blindness after Scarlatinal Nephritis with Dropsy, Forster, loco citato.

7. Forster, Graefe und Saemisch, Hahdbuch, loco citato.

8. Boston Medical and Surgical Journal, May 1, 1902.

—*Med. Fortnightly.*

